# Novel potential biomarkers for severe alcoholic liver disease

**DOI:** 10.3389/fimmu.2022.1051353

**Published:** 2022-12-13

**Authors:** Jia Huang, Jiachi Yu, Jianan Wang, Jiayu Liu, Wei Xie, Ruibing Li, Chengbin Wang

**Affiliations:** ^1^ Medical School of Chinese PLA, Beijing, China; ^2^ Department of Laboratory Medicine, The First Medical Center of Chinese PLA General Hospital, Beijing, China

**Keywords:** Alcoholic liver disease, biomarkers, liver cirrhosis, proteomics, LC-MS/MS

## Abstract

**Background:**

Alcoholic liver disease (ALD) is a leading cause of advanced liver disease; however, minor clinical symptoms in the early stage frequently result in delayed diagnosis and therapy. Invasive liver biopsy, the gold standard for diagnosing ALD, is unsuitable for repetitive analysis. This study aims to identify potential serum biomarkers that could contribute to non-invasive disease screening and monitoring.

**Methods:**

Label-free LC-MS/MS quantitative proteomics analysis was performed to identify differentially expressed proteins in the discovery cohort, followed by bioinformatics analysis based on the KEGG, GO, and String databases. Prioritized proteins were validated subsequently by quantitative assays. The area under the receiver operating characteristic curve (AUROC) was used to assess the diagnosis performance of potential biomarkers.

**Results:**

A total of 161 differentially expressed proteins were identified in the discovery cohort, of which 123 were up-regulated and 38 were down-regulated. B2M, IGFALS, and IGFBP3 were evaluated, and all demonstrated excellent diagnosis performance with AUROCs of over 0.9 when distinguishing patients with severe ALD from healthy controls. The AUROC values of B2M, IGFBP3, and IGFALS were 0.7131, 0.8877, and 0.9896 for differentiating severe ALD from non-severe ALD to indicate disease severity. B2M could distinguish patients with non-severe ALD and HC participants with an AUROC value of 0.8985. The efficiency of multiple combinations of these biomarkers was superior to that of the existing liver fibrosis evaluation indices used to monitor disease progression, with AUROC values of over 0.9. IGFALS showed a positive correlation with ALT/AST (r=0.4648, *P*=0.0009) and may be developed as a therapeutic target.

**Conclusion:**

This proteomic study identified three novel candidate proteins as promising circulating biomarkers for clinical diagnosis and disease progression and also provided the proteomic atlas for ALD pathophysiological mechanisms.

## 1 Introduction

Alcoholic liver disease (ALD) is one of the main causes of chronic liver diseases globally, with a particularly high incidence in the United States and Europe. Alcohol has harmful effects and is responsible for more than 200 diseases. Cardiovascular diseases account for the largest number of alcohol-related deaths, followed by injuries, liver cirrhosis, and cancer (1). However, alcohol-attributable scores are highest with respect to liver diseases (especially cirrhosis) and fetal alcohol syndrome (2). According to the World Health Organization (3), alcohol abuse is a risk factor for approximately 50% of cirrhosis-related deaths worldwide.

ALD begins with hepatic steatosis involving the accumulation of triglycerides in hepatocytes, followed by alcoholic hepatitis and fibrosis. Cirrhosis is observed in approximately 10% to 20% of patients with ALD, and patients with alcoholic hepatitis are at the highest risk due to the accelerated progression of fibrosis (4). Since the presence of advanced fibrosis or cirrhosis in compensated patients is a major predictor of long-term survival (5), it is clinically important to diagnose patients with advanced fibrosis before decompensation for improving survival.

Clinical diagnosis of ALD is frequently based on alcohol consumption, clinical symptoms, liver imaging, and biopsy results, excluding alternative causes of liver injury (6). However, more than 90% of patients with ALD have nonspecific symptoms or are asymptomatic (7), which renders the clinical diagnosis of ALD difficult. Even though liver biopsy is still considered the gold standard for diagnosing and assessing the stages of ALD (8), the invasive procedure is not recommended for disease screening. Numerous non-invasive tests have been developed during the last decades. Magnetic resonance imaging techniques demonstrate superior sensitivities and specificities for liver histological morphology analysis than ultrasound. However, their high costs limit their use in routine clinical practice (9). FIB-4, APRI, and FibroTest based on blood biochemical indices are commercially available; however, most of these tests are still considered auxiliary diagnostic modalities. Therefore, finding valuable biomarkers of ALD with highly sensitive technology will be of remarkable significance for monitoring disease progression, timely treatment, and exploration of underlying pathological mechanisms.

Most of the proteins secreted by the liver are released into the peripheral blood, which is easy to obtain and reflects the pathophysiological changes of the liver. Liquid chromatography-tandem Mass Spectrometry (LC-MS/MS) can demonstrate accurate quantification of small molecule proteins and peptides due to its high sensitivity and accuracy. In this study, we leverage the ALD discovery cohort to characterize the circulating proteome and reveal potential biomarkers correlated with the severity of ALD.

## 2 Methods and materials

### 2.1 Patients and serum samples

The study was approved by the Medical Ethics Committee of Chinese PLA General Hospital (S2022-451-01). Sensitive information was removed, and data were anonymized.

In total, 93 patients admitted at Chinese PLA General Hospital were enrolled in this study. Patients with ALD were diagnosed based on imaging or pathological and clinical criteria (alcohol consumption over 20 g/d for females and over 40 g/d for males, drinking history >5 years) (10, 11). The patients were divided into severe ALD and non-severe ALD cohorts by specialists according to the NAFLD activity score–clinical research network (NAS–CRN) (12). Patients with ALD were assigned a fibrosis (F0–4) score, and we defined non-severe ALD as F0-3 and severe ALD as >F4 for subsequent analysis. Patients with any metabolic diseases, cancer, coronary heart disease, viral hepatitis, drug-induced liver disease, autoimmune liver disease, or contact with infected water were excluded. The severe ALD group included patients with decompensated alcoholic cirrhosis (n=46). The non-severe ALD group included patients with alcoholic hepatic steatosis (n=6) and patients with compensated alcoholic cirrhosis (n=7). Healthy controls (HC) (n=34) had normal biochemical indicators and showed normal imaging results of the liver, gallbladder, and spleen. Furthermore, the participants did not report heavy drinking history, cancer, or any metabolic diseases.

A total of 18 participants with similar demographic characteristics (nine patients with severe ALD and nine HC) were enrolled as a discovery cohort to screen the differential protein profiles between ALD and HC. To validate potential proteins of the discovery cohort, we included 13 patients with non-severe ALD (six alcoholic hepatic steatosis and seven compensated alcoholic cirrhosis), 37 patients with severe ALD, and 25 HC who met the inclusion criteria as a validation cohort. Concentrations of serum potential biomarkers in the validation cohort were measured by enzyme-linked immunosorbent assay (ELISA) and turbidimetric inhibition immunoassay, and the diagnostic performance of potential biomarkers was further evaluated by receiver operating characteristic (ROC) curves.

Fast blood samples were collected in serum-separating tubes, centrifuged at 3000 g for 15 min within 120 min of collection, and the supernatant was aliquoted and stored at −80°C until further analysis.

### 2.2 Acquisition and processing of proteomic data

#### 2.2.1 Sample preparation

Serum samples stored at -80°C were removed and thawed to determine the serum protein concentration using Nanodrop. Samples were incubated in the top 14 abundant protein depletion mini spin columns (A36370, Thermo Fisher, USA) for 30 min. The liquids were subsequently centrifuged in 10kD Fasp tubes (UFC501024, Millipore, USA). The disulfide bond was destroyed in 10 mM dithiothreitol (DTT), which was prevented from closing again by treatment with 50 mM iodoacetamide (IAA), followed by digestion with trypsin (protein: trypsin = 50:1) overnight (>12h) at 37°C. Subsequently, the reaction was terminated using 5% formic acid (FA), and the peptides were loaded on the C18 film for desalting with 0.1% FA. After vacuum drying, the peptides were re-dissolved with 0.1% FA, and 8.8 µL was obtained for analysis. The details of serum protein concentration and the quality of each sample for the following analysis have been listed in **Table 1**.

**Table 1:** Serum protein concentration and quality for analysis.

	Serum protein concentration (mg/ml)	Peptide concentration (mg/ml)	Volume for LC-MS/MS analysis (ul)
HC1	63.89	0.217	2
HC2	59.32	0.371	1
HC3	60.29	0.272	1.5
HC4	64.92	0.334	1.2
HC5	65.23	0.228	1.8
HC6	49.34	0.250	1.5
HC7	52.70	0.400	1
HC8	58.24	0.381	1
HC9	68.89	0.226	1.8
ALD1	62.39	0.235	1.5
ALD2	59.94	0.164	2.5
ALD3	51.27	0.228	1.8
ALD4	58.28	0.133	3
ALD5	54.38	0.169	2.2
ALD6	63.43	0.249	1.5
ALD7	64.27	0.233	1.5
ALD8	50.29	0.106	2.5
ALD9	47.69	0.108	3.5

#### 2.2.2 Data acquisition

The LC-MS/MS Analysis of peptides was performed using a quadrupole Orbitrap mass spectrometer combined with the Ultimate-3000 HPLC system. Peptides were dissolved partially in phase A [2% acetonitrile (ACN) + 98% H_2_O + 0.1% FA] to detect the protein sequence. A total of 300–500 ng of peptides was separated using an in-house C18 analytical column and measured with increasing concentrations of phase B [80% acetonitrile (ACN) + 20% H_2_O + 0.1% FA]. Data acquisition of the mass spectrometer was performed in data-dependent acquisition (DDA) mode.

#### 2.2.3 Bioinformatic analysis

The Q-Exactive raw files were exported and identified in the Uniprot database (Homo sapiens 2020) to calculate protein peak areas of all samples using Thermo Proteome Discoverer (PD) V2.2.0.388. For further analysis, the data corresponding to the peak areas were imported in Perseus V2.0.6.0 to filter proteins with missing values exceeding 70% according to the proportion of missing values in each group. The missing values were replaced from the normal distribution, and 345 genes were analyzed in 18 samples. The student’s *t* test and Z-score normalization were performed for subsequent analyses. Differentially expressed proteins were screened *via* a two tails student’s *t*-test with *q*<0.05 and a fold change (FC) >1.5 or <0.67. Volcano plots, heatmaps, and principal component analysis (PCA) were performed using pre-processed data with OmicStudio and Oebiotech online tools. Gene ontology (GO) analysis and Kyoto encyclopedia of genes and genomes (KEGG) database evaluation were conducted using the Database for Annotation, Visualization, and Integrated Discovery (https://cloud.oebiotech.cn; http://vip.SangerBox.com) to identify enriched proteins.

### 2.3 Validation of selected protein expression

To validate the proteomic results, the following three proteins were selected: IGFBP3, IGFALS, and B2M. The concentration of B2M in serum was measured by turbidimetric inhibition immunoassay using SIEMENS BN II (Germany). IGFBPP3 ELISA kits (CSB-E04590h, CUSABIO, China) and IGFALS ELISA kits (EH3259, Fine Test, China) were used for evaluation according to the manufacturer’s instructions.

Briefly, 100 μl of each sample was added and incubated for 90–120 minutes at 37°C. The liquid was removed, and 100 μl of biotinylated antibody (1x) was added to each well followed by incubation for 60 minutes at 37°C. Then, aspirate each well and wash it thrice. A total of 100 μl of horse radish peroxidase (HRP)‐avidin (1×) was added to each well, followed by incubation for 30–60 minutes at 37°C. Subsequently, 90 μl of TMB substrate was added and incubated for 15 minutes at 37°C; the samples were shielded from light. Finally, 50 μl of stop Solution was added to each well, and the optical density was determined at 450 nm within five minutes. Measurements were repeated thrice.

### 2.4 Statistical analysis

Statistical analysis was performed using SPSS 26.0 and Graphpad Prism software. The Shapiro-Wilk test was used to analyze the normality of data distribution. Continuous variables conforming to the normal distribution were expressed as mean ± standard and evaluated by *t* test or analysis of variance (ANOVA). Variables without normal distribution were expressed as median M (quartiles Q1, Q3) and analyzed using the Mann-Whitney *U* test or Kruskal-Wallis *H* test. To evaluate the diagnostic performance of the screened potential biomarkers, ROC curves were derived. The area under the ROV curve (AUROC), sensitivity, and specificity were identified according to the Youden index, which was used as accuracy criteria to assess the diagnosis performance. Logistic regression analysis was used to assess the diagnostic accuracy of combined biomarkers. Results with two-tailed *P*<0.05 were considered statistically significant.

## 3 Results

### 3.1 Clinical characteristics of the study cohorts

Overall, 93 samples from two cohorts (samples: discovery cohort, 18; validation cohort, 75) were included in the study. The detailed demographic data and clinical characteristics are summarized in **Tables 2**, **3**. The median age ranged from 37.1 to 53.7 years, and the patient cohort was predominantly male.

**Table 2:** Clinical characteristics of participants in the discovery cohort.

	Severe ALD (n=9)	Health control (n=9)	*P* value
Age	42.72 ± 5.31	37.18 ± 2.37	0.14
Gender	men	men	/
ALT (U/L)	16.90 (13.65, 22.50)	12.20 (10.40, 16.85)	0.171
AST (U/L)	24.80 (16.55, 36.40)	16.22 ± 2.86	0.047
ALB (g/L)	38.23 ± 4.02	47.18 ± 1.89	0.014
Tbil (µmol/L)	17.20 (7.45, 34.95)	11.43 ± 3.47	0.251
ALP (U/L)	88.04 ± 25.42	70.20 (62.35, 97.30)	0.310
GGT (U/L)	80.84 ± 94.17	13.00 ± 4.08	0.001
PLT (10^9^/L)	83 ± 49	230 ± 38	0.001
APRI	0.34 ± 0.21	0.07 ± 0.02	0.051
FIB-4	4.82 ± 2.30	0.65 ± 0.14	0.04
AAR	1.69 ± 0.63	1.20 ± 0.32	0.085

**Table 3:** Clinical characteristics of participants in the validation cohort.

	Non-severe ALD (n=13)	Severe ALD (n=37)	Health control (n=25)	*P* value
Age	46.31 ± 8.85	53.78 ± 8.58	39.76 ± 8.66	<0.001
Gender	male	male	male	/
ALT (U/L)	32.80 (17.55, 69.05)	19.00 (13.00, 31.00)	19.40 (13.10, 21.40)	0.036
AST (U/L)	26.80 (19.15, 55.95)	28.60 (19.60, 47.80)	16.50 (13.50, 18.80)	<0.001
ALB (g/L)	46.10 (33.83, 47.83)	33.00 (29.20, 40.20)	48.30 (47.23, 49.43)	<0.001
Tbil (µmol/L)	13.10 (9.85, 16.50)	26.30 (15.60, 52.20)	12.20 (8.75, 13.00)	<0.001
ALP (U/L)	76.80 (65.40, 133.55)	89.45 (70.98, 131.40)	74.80 (65.15, 88.00)	0.054
GGT (U/L)	97.20 (34.05, 300.40)	40.40 (27.80, 76.80)	18.30 (14.02, 28.70)	<0.001
TC (mmol/L)	3.8 ± 1.1	2.9 ± 0.9	4.0 ± 0.8	0.002
PLT (10^9^/L)	211 (132, 271)	75 (56, 114)	262 (218, 301)	<0.001
APRI	0.17 (0.15, 0.38)	0.40 (0.32, 0.70)	0.06 (0.05, 0.08)	<0.001
FIB-4	1.23 (0.81, 2.25)	5.31 (3.05, 6.81)	0.52 (0.39, 0.68)	<0.001
AAR	0.64 (0.58, 1.26)	1.68 (1.03, 2.14)	0.92 (0.78, 1.04)	<0.001

ALB, albumin; Tbil, total bilirubin; GGT, gamma-glutamyltransferase; TC, total cholesterol; PLT, platelet count; APRI, AST to platelet ratio index; FIB-4, fibrosis-4 index: age (years) × AST [U/L]/(platelets [10^9^/L] × (ALT [U/L])^1/2^); AAR, aspartate aminotransferase-to-alanine aminotransferase ratio.

*P value of severe ALD group compared to non-severe ALD group. ALT *P*=0.041; AST *P*=0.745; ALB *P*=0.007; Tbil *P*=0.001; ALP *P*=0.301; GGT *P*=0.117; TC *P*=0.029; PLT *P*<0.001; APRI *P*=0.002; FIB4 *P*<0.001; AAR *P*=0.006.

*P* value of severe ALD group compared to HC group. ALT *P*=0.509; AST *P*<0.001; ALB *P*<0.001; Tbil *P*<0.001; ALP *P*=0.014; GGT *P*<0.001; TC *P*=0.001; PLT *P*<0.001; APRI *P*=0.002; FIB4 *P*<0.001; AAR *P*<0.001.

*P* value of non-severe ALD group compared to HC group. ALT *P*=0.010; AST *P*<0.001; ALB *P*=0.009; Tbil *P*=0.178; ALP *P*=0.517; GGT *P*<0.001; TC *P*=0.859; PLT *P*=0.075; APRI *P*=0.002; FIB4 *P*=0.001; AAR *P*=0.681.

Clinical data were obtained from the medical records. Enrolled patients with severe or non-severe ALD had over 40 g/d alcohol intake and more than five years of drinking history. In the discovery cohort, no significant differences were observed with respect to most serum biochemical indices between patients with ALD and controls as well as in age (*P*<0.05) between groups.

### 3.2 Differentially expressed proteins of discovery cohort based on LC-MS/MS

**Figure 1:**
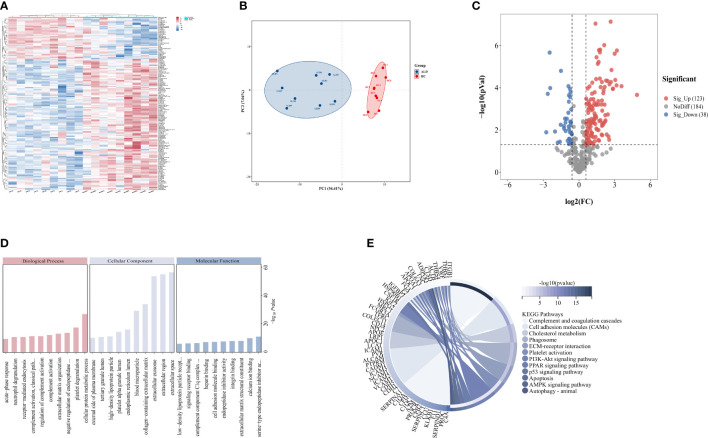
Differential expressed proteins of clinical patients. **(A)** Heatmap represents gene expression trends in HC and ALD groups. **(B)** PCA shows two dimensionalities of HC and ALD groups according to differential proteins. **(C)** Volcano plot shows the identified proteins of HC and ALD groups distribution. **(D)** GO enrichment shows function and location of differential proteins. **(E)** KEGG enrichment shows the related pathways with *P*< 0.05.

A total of 345 proteins were identified by label-free LC-MS/MS proteomic analyses in serum samples derived from patients with severe ALD and HC (*P*<0.05 and |FC| >1.5) (**Figures 1A-C**). A total of 161 differentially expressed proteins were visualized in a volcano plot with 123 significantly upregulated proteins shown in red and 38 significantly downregulated proteins shown in blue (**Figure 1C**; details of the top 20 differentially expressed proteins are provided in **Table 4**). A heatmap and PCA results showed distinct clustering of patients with severe ALD separately from HC (**Figures 1A, B**).

**Table 4:** Top 20 differentially expressed proteins.

Description	Accession	*P* Value	Fold Change	Log2FC
**UP-regulation**				
CRP	P02741	<0.001	29.44764	4.88008
ICAM1	P05362	<0.001	11.95158	3.57913
CD5L	O43866	<0.001	9.120545	3.18912
NPC2	G3V3D1	<0.001	8.92839	3.1584
MRC1	P22897	<0.001	8.288724	3.05115
ADA2	Q9NZK5	<0.001	7.998281	2.99969
ENPP2	E7EUF1	<0.001	7.270578	2.86207
B2M	P61769	<0.001	7.196772	2.84735
CD163	Q86VB7	<0.001	6.653576	2.73413
PLXDC2	Q6UX71	<0.001	6.433617	2.68563
**Down-regulation**				
C4A	P0C0L4	<0.001	0.148115	-2.75521
CNDP1	Q96KN2	<0.001	0.161839	-2.62737
IGFALS	P35858	<0.001	0.180848	-2.46715
IGFBP3	P17936	<0.001	0.18657	-2.42221
DBH	P09172	0.012	0.247363	-2.0153
HP	P00738	0.039	0.289369	-1.78902
SEMG2	Q02383	0.036	0.328456	-1.60623
APOC3	B0YIW2	0.006	0.343938	-1.53978
IGF2	P01344	<0.001	0.34887	-1.51924
GAPDH	P04406	0.007	0.416616	-1.26321

GO annotation (**Figure 1D**) and KEGG pathway enrichment (**Figure 1E**) were performed to reveal relevant functional characteristics and biological information of significantly differentially expressed proteins in patients with severe ALD. The main cellular components involved were blood microparticles, platelet granule lumen, and the endoplasmic reticulum lumen. Biological processes were mainly involved in processes such as neutrophil degranulation, complement activation, and acute phase reaction. The main molecular functions were signaling receptor binding, lipid transport, peptide inhibitor activity, and antioxidant activity. Among total pathways enriched in the KEGG database, the top 30 pathways with the highest significance included processes such as complement and coagulation cascade, cholesterol metabolism, ECM signaling pathway, and autophagy.

Besides, proteomics analysis without abundant protein were also performed on the same 18 samples to find out potential biomarkers which could be easier to test and apply in clinics (**Supplementary Figure S1**). Among the top 20 proteins with the largest difference within the two methods, B2M, IGFBPP3, IGFALS and CRP were overlapped significant differential proteins (**Supplementary Table S1**), which would be targets for validation.

### 3.3 Identification of potential biomarkers in the validation cohort

**Figure 2:**
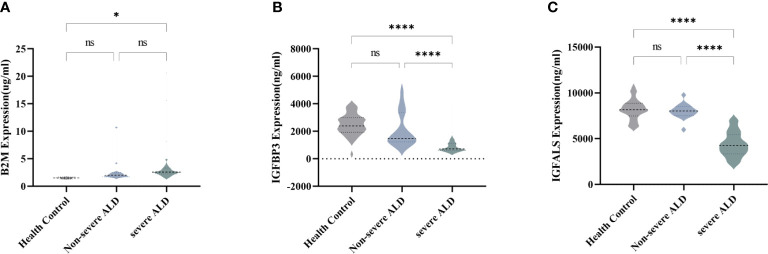
Serum concentration of B2M, IGFBP3, and IGFALS in different stages of ALD patients. **(A)** Serum concentration of B2M in HC group, non-severe ALD group, and severe ALD group. **(B)** Serum concentration of IGFBP3 in HC group, non-severe ALD group, and severe ALD group. **(C)** Serum concentration of IGFALS in HC group, non-severe ALD group, and severe ALD group. ns is *P*>0.05; * *P*<0.05; ****P<0.0001.

To verify the expression of the four potentially promising biomarkers in different stages of ALD, 75 participants were classified into a validation cohort, including the non-severe ALD group (n=13), severe ALD group (n=37), and HC group (n=25). The blood CRP levels of patients in each group and found that there was no significant difference in CRP levels among three groups (**Supplementary Figure S2**). Three potential proteins, namely, B2M, IGFBP3, and IGFALS, showed the most significant differences and were further analyzed using quantitative assays. For these three biomarkers, no significant differences were observed in patients with non-severe ALD compared to those observed in the HC, whereas the changes were distinct when compared to the results of the severe ALD group (*P*<0.05) (**Figure 2**). Consistent with the results of the discovery cohort, the expression of B2M in the validation cohort was significantly increased in the severe ALD group (**Supplementary Table S4**), while the protein expression of IGFBP3 and IGFALS was notably decreased in the severe ALD group (*P*<0.05). The expression of IGFBP3, and IGFALS in the non-severe group was substantially different from that in the severe ALD group.

### 3.4 Evaluation of the diagnostic performance of B2M, IGFBP3, and IGFALS as potential markers

To identify potential biomarkers associated with severe ALD that would distinguish patients with severe ALD from those with non-severe ALD and HC, the model performance of B2M, IGFBP3, and IGFALS was evaluated independently and in combination and was benchmarked against commercially available serum tests for evaluation of liver fibrosis FIB-4 index, AAR, and APRI.

**Figure 3:**
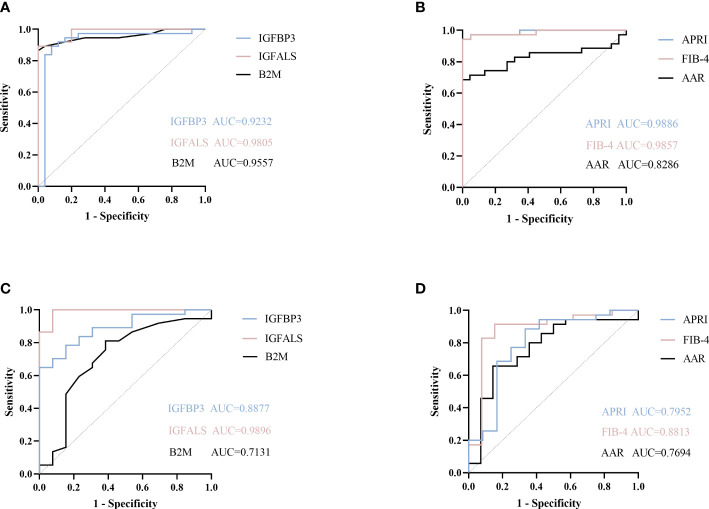
ROC curve of potential proteins. **(A)** ROC curve of B2M, IGFBP3, and IGFALS to diagnose severe ALD from HC group. **(B)** ROC curve of APRI, FIB-4, AAR to diagnose severe ALD from HC group. **(C)** ROC curve of B2M, IGFBP3, and IGFALS to diagnose severe ALD from non-severe ALD group. **(D)** ROC curve of APRI, FIB-4, AAR to diagnose severe ALD from non-severe ALD group.

According to ROC curves shown in **Figure 3**, for differentiating patients with severe ALD from HC, the AUROC of B2M was 0.9557 (*P*<0.001, sensitivity: 89.19%, specificity: 96%), that of IGFBP3 was 0.9232 (*P*<0.001, sensitivity: 91.89%, specificity: 88%), and that of IGFALS was 0.9805 (*P*<0.001, sensitivity: 94.59%, specificity: 80%). In distinguishing patients with non-severe ALD from HC, the AUROC of B2M was 0.8985 (*P*<0.001, sensitivity: 84.62%, specificity: 72%); however, the values of IGFBP3 and IGFALS did not show any significant differences (*P*>0.05). In differentiating severe ALD from non-severe ALD, the AUROCs of B2M, IGFBP3 and IGFALS were 0.7131 (*P*=0.0234, sensitivity: 81.08%, specificity: 61.54%), 0.8877 (*P*<0.001, sensitivity: 70.27%, specificity: 92.31%), and 0.9896 (*P*<0.001, sensitivity: 100%, specificity: 92.31%), respectively. The ROC curves of CRP were calculated as independent diagnostic model with *P*>0.05 and listed in **Supplementary Tables S2**, **S3**.

The FIB-4 index, AAR, and APRI have been widely used as non-invasive indicators of liver fibrosis, relying on age and biochemical indicators to determine the degree of fibrosis (13, 14). In this study, the AUROC of these three indicators was calculated and compared to evaluate the diagnostic efficacy of B2M, IGFBP3, and IGFALS. The results showed that the AUROC values of FIB-4, APRI, B2M, IGFBP3, and IGFALS for distinguishing patients with severe ALD from HC were more than 0.85, reflecting a strong diagnostic efficacy. However, the diagnostic performance of AAR with an AUROC value of 0.8286 was slightly weaker. While distinguishing patients with non-severe ALD from HC, the AUROC values of APRI and FIB-4 were higher than 0.85 (*P*<0.05), whereas values for AAR showed no significant differences (*P*>0.05). However, B2M showed better diagnostic performance than APRI and FIB-4. While differentiating the severe ALD group from the non-severe ALD group, IGFBP3 and IGFALS showed better independent diagnosis performance than FIB-4 (AUROC 0.8813), AAR (AUROC 0.7694), and APRI (AUROC 0.7952) (**Table 5**).

**Table 5:** Characteristics of potential proteins ROC curves.

		AUROC	95% CI	Sensitivity	Specificity	*P* value
	
**HC-SA**						
	B2M	0.9557	0.9043 to 1.000	0.8919	0.9600	<0.0001
	IGFBP3	0.9232	0.8344 to 1.000	0.9189	0.8800	<0.0001
	IGFALS	0.9805	0.9553 to 1.000	0.9459	0.8000	<0.0001
	APRI	0.9886	0.9668 to 1.000	0.9714	0.9500	<0.0001
	FIB-4	0.9857	0.9589 to 1.000	0.9429	1	<0.0001
	AAR	0.8286	0.7175 to 0.9397	0.7143	0.9545	<0.0001
**HC-HSA**						
	B2M	0.8985	0.7995 to 0.9974	0.8462	0.7200	<0.0001
	IGFBP3	0.6431	0.4189 to 0.8672	0.5385	0.9200	0.1525
	IGFALS	0.5631	0.3749 to 0.7513	0.4615	0.7200	0.5282
	APRI	0.8917	0.7516 to 1.000	0.8333	0.9500	0.0003
	FIB-4	0.8731	0.7403 to 1.000	0.8462	0.8500	0.0004
	AAR	0.5455	0.3090 to 0.7819	0.5000	0.9545	0.6496
**SA-NSA**						
	B2M	0.7131	0.5361 to 0.8901	0.8108	0.6154	0.0234
	IGFBP3	0.8877	0.7972 to 0.9783	0.7027	0.9231	<0.0001
	IGFALS	0.9896	0.9668 to 1.000	1.0000	0.9231	<0.0001
	APRI	0.7952	0.6297 to 0.9608	0.8857	0.6667	0.0025
	FIB-4	0.8813	0.7530 to 1.000	0.9143	0.8462	<0.0001
	AAR	0.7694	0.6160 to 0.9228	0.6571	0.8571	0.0035

NSA, Non-severe ALD; SA, Severe ALD; HC, Health controls.

To further improve the diagnosis performance, novel biomarkers were combined to develop models for diagnosis. B2M and IGFBP3 were combined as the diagnostic model A, B2M and IGFALS as model B, and IGFALS and IGFBP3 as model C, and the combination of these three markers was characterized as model D. The AUROC values of multiple combinations were calculated by logistic regression analysis, yielding the sensitivity, specificity, and corresponding predictive values. The AUROC values of models A, B, and D were above 0.9, showing significant efficiency to differentiate patients with non-severe ALD from HC with high sensitivity and specificity (**Table 6**). The efficiency was better than APRI, FIB-4, and AAR. Given the AUROC values of model C, IGFBP3 and IGFALS showed poor diagnostic efficacy for HC and patients with non-severe ALD. As shown by the results of model D, the inclusion of IGFALS could slightly improve the diagnostic efficacy of model A. Hence, model A could be an optimal monitoring index for non-severe ALD. The AUROC values of four diagnostic models were all above 0.9, demonstrating significant efficiency to differentiate patients with severe ALD from HC and those with non-severe ALD with high sensitivity and specificity (**Table 7**). Compared with APRI, FIB-4, and AAR, these four models had better diagnosis performance. Given the possibility of overfitting, which resulted in model D having the same AUROC, sensitivity, and specificity values as model C, model C was selected as the optimal monitor index for severe ALD in our study.

**Table 6:** Characteristics of diagnostic models ROC curves for non-severe ALD.

		AUROC	95%CI	Sensitivity	Specificity	*P* value
**Model A**	B2M-IGFBP3	0.9354	0.8527 to 1.000	0.9231	0.8400	<0.0001
**Model B**	B2M-IGFALS	0.9015	0.8061 to 0.9970	0.9231	0.7200	<0.0001
**Model C**	IGFBP3-IGFALS	0.5908	0.3854 to 0.7962	0.6154	0.6800	0.3640
**Model D**	B2M-IGFBP3-IGFALS	0.9385	0.8630 to 1.000	0.8462	0.9200	<0.0001

**Table 7:** Characteristics of diagnostic models ROC curves for severe ALD.

		AUROC	95% CI	Sensitivity	Specificity	*P* value
Model A	B2M-IGFBP3	0.9168	0.8384 to 0.9953	0.8649	1	<0.0001
Model B	B2M-IGFALS	0.9896	0.9668 to 1.000	0.8919	0.9231	<0.0001
Model C	IGFBP3- IGFALS	0.9958	0.9848 to 1.000	0.9459	1	<0.0001
Model D	B2M-IGFBP3-IGFALS	0.9958	0.9848 to 1.000	0.9459	1	<0.0001

### 3.5 IGFALS correlated with ALT/AST

**Figure 4:**
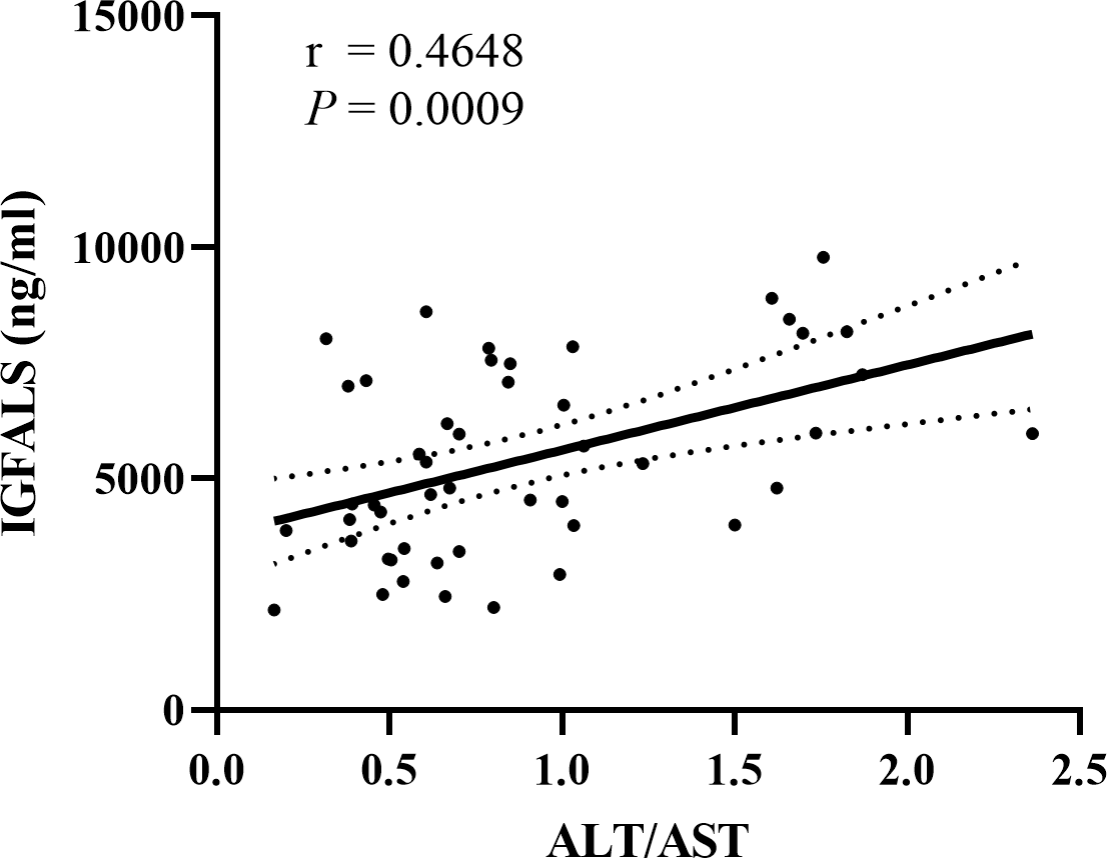
Correlation between IGFALS expression with ALT/AST in ALD patients. Pearson correlation coefficient of IGFALS is r=0.4648 (*P*=0.0009) showing a positive correlation with ALT/AST represented by enrolled samples.

To detect if these three genes could be considered therapeutic targets for ALD, we analyzed the correlation between the expression of B2M, IGFBP3, and IGFALS with ALT/AST in patients with ALD, respectively. Since the quantitative data involved were normally distributed, linear correlation analysis was performed. Pearson’s correlation coefficient r represents the correlation degree of the two indicators, and the *P* value determines the presence of a linear correlation between the two indicators. Pearson correlation coefficient of B2M (r=-0.0432, *P*=0.7706) and IGFBP3 (r=0.07411, *P*=0.6166) showed an absence of correlation between B2M expression and IGFBP3 expression with ALT/AST. The results of IGFALS (r=0.4648, *P*=0.0009) showed a positive correlation with ALT/AST, as represented by the samples included in this study (**Figure 4**).

## 4 Discussion

ALD is a major cause of cirrhosis and liver failure, which progresses from steatosis and steatohepatitis. Liver fibrosis is widely considered to be a dynamic process with regression potential (15). However, the hypothesis that cirrhosis could reverse to a completely normal liver architecture remains controversial because the formation of non-reducible crosslinked collagen inhibits extracellular matrix (ECM) remodeling and degradation (16, 17). Additionally, shared pathophysiological features, such as immune dysfunction and coagulation disturbance, of compensated and decompensated alcoholic cirrhosis may be observed early and contribute to disease progression (18). Hence, monitoring patients with excessive daily alcohol consumption to assess liver function promptly is critical for optimal disease management.

Liver biopsy is still the primary diagnostic and classification criterion for ALD (19), but repetitive invasive tests are difficult to use as a screening tool in routine practice. Clinical liver enzyme indicators as auxiliary diagnostic modalities scarcely indicate the development of ALD (19). Circulating proteome could directly reflect the protein alterations associated with ALD, particularly severe ALD. Developing circulating biomarkers based on highly sensitive tools may help in non-invasive screening, disease progression monitoring, and prognostic assessment of ALD patients, and provide new insights into the underlying pathogenesis.

In this study, label-free LC-MS/MS results showed that differential proteins were primarily involved in processes such as lipid metabolism, complement and coagulation cascade, immune system, and blood flow transport. The involvement of differential proteins involved in inflammation and immune system regulation may be attributed to increased immune cell infiltration and global systemic inflammation, ECM remodeling and scar tissue formation in liver fibrosis, and tissue leakage (20). Enriched pathways analyzed *via* the KEGG database further reflected the pathogenesis of ALD, such as ECM receptor interaction and local adhesion spots, which play important roles in the development of liver fibrosis (21, 22). Insulin resistance, inflammation, oxidative stress, and mitochondrial dysfunction observed in the GO and KEGG enrichment analyzed accelerated the progression of alcohol-related hepatitis to advanced stages of ALD (23). The Ras/MAPK/ERK pathway impaired liver regeneration by blocking insulin signaling and increasing cell remodeling, DNA damage, and mitochondrial dysfunction *via* the PI3K-Akt pathway-related signaling (24, 25). These pathways could provide new directions for further research on ALD.

B2M, IGFBP3, and IGFALS were promising targets in serum differential proteomic analysis. We identified these three proteins in the validation cohort *via* quantitative ELISA and turbidimetric inhibition immunoassay. Notably, a remarkable difference in serum concentrations of these three proteins was observed between different stages of ALD, suggesting that their expression may be correlated with ALD progression. Besides, their ROC curves illustrated that they showed independent diagnosis performance for distinguishing patients with severe ALD from HC, which was comparable with the recognized FIB-4, APRI, and AAR tests. The diagnosis efficiency of B2M was better than that of the three recognized indicators when distinguishing non-severe ALD from HC. Multiple combinations of B2M, IGFBP3, and IGFALS showed excellent AUROC values with superior sensitivities and specificities for distinguishing severe ALD from non-severe ALD than other biological tests. Among four combined diagnosis models designed in this study, model C (IGFBP3 combined IGFALS) was considered to be the optimal choice to differentiate severe ALD from HC and non-severe ALD, avoiding the probable overfitting of model D. IGFALS showed a positive correlation with ALT/AST, and hence, might be developed as a therapeutic target.

Accumulating data suggest that B2M is involved in a wide range of physiological and pathological functions, such as cell proliferation and apoptosis, and is also regarded as an important prognostic factor and predictor of survival associated with multiple cancers. B2M activates the PI3K/AKT/mTOR signaling pathway by promoting secretion of transforming growth factor-β1 (TGFβ1) (26). The CRISPR-Cas9 system was used to develop CAR T cells with three types of gene editing, including that in the B2M. The CAR T cells were injected into the brain, and prolonged survival of mice harboring intracranial tumors was observed (27). However, the role of B2M in metabolic liver disease remains primarily unelucidated. Luo (28) et al. found that B2M, one of the components of HLA class I, could be used as a downstream protein to activate autophagy and apoptosis during hepatotoxicity in hepatocytes. These findings suggested that upregulation of the expression of B2M may have detrimental effects on liver health. B2M was also reported to participate in the neuroimmune regulation of alcohol consumption (29). Increased levels of B2M may serve as a potential biomarker of the severity of ALD, as proposed for HCV cirrhosis and carcinoma (30).

We also identified IGFBP3 and IGFALS as promising biomarkers of ALD severity. IGFBP3 predominantly constitutes circulating forms of insulin-like growth factors (IGFs). It regulates IGF functions and also possesses IGF-independent roles to modulate cell growth and survival (31). Low levels of IGFBP3 are tightly associated with the development of common malignancies. Our data showed that the expression of IGFBP3 was decreased in patients with ALD and was correlated with the severity. This finding was consistent with the results of previous studies (32, 33) that showed that IGFBP3 was the most pronounced downregulated protein in patients with Child-Pugh C. A decrease in plasma IGFBP3 levels has also been observed in patients with other chronic liver disease-non-alcoholic fatty liver disease (NAFLD) (34). Another large multicenter study provided additional information on IGFBP3 as an independent prognostic value associated with ALD survival (35), and lower levels were associated with worse outcomes in patients with cirrhosis, representing a promising prognostic tool. However, contrasting results were observed in other studies, which showed that increased expression of IGFBP3 in patients with alcoholic hepatitis could directly promote lipid droplet formation facilitating ethanol-induced steatosis. Additionally, hepatic stellate cell-derived IGFBP3 increases lipogenesis *via* integrin receptor/Src-kinase signaling and p-Akt up-regulation in primary hepatocytes, thereby contributing to ethanol-induced steatosis (36). *In vivo* data showed that IGFBP3 is a novel effector molecule and not just a “binding protein” with IGF-independent actions on metabolism and cell growth. Furthermore, it is associated with hepatic insulin resistance and decreased peripheral glucose sensitivity (37). In summary, clinical data for IGFBP3 is contrasting, and IGFBP3 possibly has more than one role in the disease. Further studies may be needed to elucidate its biological function in the spectrum of alcoholic liver diseases.

Despite the fact that transcriptional analysis of liver IGFALS to examine its role in liver diseases is rarely performed, IGFALS forms ternary complexes with IGF-I and IGFBP3 in the circulation and regulates body growth, development, and other physiological/pathophysiological processes (38). The down-regulation of IGFALS transcription/translation leads to a mitochondrial decline and impairment of cellular regeneration *via* the growth hormone/insulin-like growth factor (GH/IGF) axis (39, 40). Chidozie J et al. demonstrated growth retardation caused by the downregulation of IGFALS in mice (41). Therefore, the down-regulation of IGFALS protein expression may indicate dysfunctional liver regeneration ability to a certain extent. In patients with ALD, injured hepatocytes cannot regenerate in time, and they are replaced by collagen, resulting in liver fibrosis. Our data revealed a decrease in the expression of IGFALS in patients with alcoholic liver fibrosis, as shown in HBV‐related hepatocellular carcinoma (HCC) (42, 43). Additionally, a positive correlation has been observed between IGFALS with ALT/AST. Serum levels of ALT and AST have been considered markers of liver injury, suggesting concomitant infection, inflammation, and coagulopathy (44). In a prior study, the ALT/AST ratio, rather than ALT or AST alone, was correlated with the degree of liver fat in the liver biopsy (45). As a liver tissue-specific gene, the down-regulation of the expression of IGFALS suggested a poor prognosis of hepatocellular carcinoma (46). These results indicate that IGFALS as a therapeutic target of ALD could demonstrate the severity and prognosis of fibrosis. The association between IGFALS and ALD may be attributed to IGF homogeneity and hormone sensitivity. The novel role of IGFALS in ALD needs further validation and illustration.

Our study has several limitations. First, our small sample size may limit the assessment of biomarker performance and necessitates a large cohort study to further validate the results. Despite our limited sample size, we still believe that our results were reliable because various confounding factors had been excluded in the early screening of differential proteins, and age difference had no significant effect on the biomarkers. We did not obtain all the liver biopsy results of all patients, and hence, could not score each patient. If patients were classified according to liver biopsy results and other controls with cirrhosis attributed to other factors were included in our cohort, we could have explored the association between potential biomarkers and the ALD disease more precisely.

Despite these limitations, our study still indicates that B2M, IGFBP3, and IGFALS are promising for ALD diagnosis and disease progression monitoring. These novel biomarkers could hopefully complement non-invasive methods for assessing liver cirrhosis to offer appropriate follow-up measures and help elucidate the underlying ALD pathogenesis.

## Data availability statement

The datasets presented in this study can be found in online repositories. The names of the repository/repositories and accession number(s) can be found below: http://www.proteomexchange.org/,PXD036941.

## Ethics statement

The studies involving human participants were reviewed and approved by Medical Ethics Committee of Chinese PLA General Hospital (S2022-451-01). Written informed consent for participation was not required for this study in accordance with the national legislation and the institutional requirements.

## Author contributions

JH carried out the experiments, analyzed data, and wrote the manuscript. JY and WX assisted with sample collection and processing. JW and JL provided technical support. RL and CW conceptualized, designed the study, and reviewed the manuscript to ensure the accuracy and authenticity of this study. The authors read and approved the final manuscript.
