# Supplementary material for: Novel potential biomarkers for severe alcoholic liver disease
Source: Front Immunol. 2022 Dec 13;13:1051353. doi: 10.3389/fimmu.2022.1051353 (PMC9794087; doi:10.3389/fimmu.2022.1051353)
Supplement: Supplementary file 1 [file DataSheet_1.docx]

Supplementary Table S1 Top 20 Differentially expressed proteins

|  | proteomic  without abundant protein depletion | | |  | proteomic  with abundant protein depletion | | |
| --- | --- | --- | --- | --- | --- | --- | --- |
|  | Description | *P* value | Fold Change |  | Description | *P* value | Fold Change |
| UP-regulation |  |  |  |  |  |  |  |
|  | PIGR | <0.001 | 13.54 |  | CRP | <0.001 | 29.44764 |
|  | B2M | <0.001 | 6.00 |  | ICAM1 | <0.001 | 11.95158 |
|  | LGALS3BP | <0.001 | 4.95 |  | CD5L | <0.001 | 9.120545 |
|  | IGKV1-9 | <0.001 | 4.31 |  | NPC2 | <0.001 | 8.92839 |
|  | IGHA2 | <0.001 | 3.89 |  | MRC1 | <0.001 | 8.288724 |
|  | CRP | <0.001 | 3.68 |  | ADA2 | <0.001 | 7.998281 |
|  | EFEMP1 | <0.001 | 3.52 |  | ENPP2 | <0.001 | 7.270578 |
|  | IGHV1-18 | <0.001 | 3.49 |  | B2M | <0.001 | 7.196772 |
|  | IGLV4-69 | <0.001 | 3.40 |  | CD163 | <0.001 | 6.653576 |
|  | FCGR3A | <0.001 | 3.31 |  | PLXDC2 | <0.001 | 6.433617 |
| Down-regulation |  |  |  |  |  |  |  |
|  | IGFALS | <0.001 | 0.052 |  | C4A | <0.001 | 0.148115 |
|  | APOF | <0.001 | 0.17 |  | CNDP1 | <0.001 | 0.161839 |
|  | APOC4 | <0.001 | 0.20 |  | IGFALS | <0.001 | 0.180848 |
|  | IGFBP3 | <0.001 | 0.24 |  | IGFBP3 | <0.001 | 0.18657 |
|  | BCHE | <0.001 | 0.24 |  | DBH | 0.012 | 0.247363 |
|  | THBS1 | <0.001 | 0.26 |  | HP | 0.039 | 0.289369 |
|  | PROC | <0.001 | 0.27 |  | SEMG2 | 0.036 | 0.328456 |
|  | PF4 | <0.001 | 0.28 |  | APOC3 | 0.006 | 0.343938 |
|  | HPR | <0.001 | 0.28 |  | IGF2 | <0.001 | 0.34887 |
|  | PRKAG2 | 0.0087 | 0.31 |  | GAPDH | 0.007 | 0.416616 |


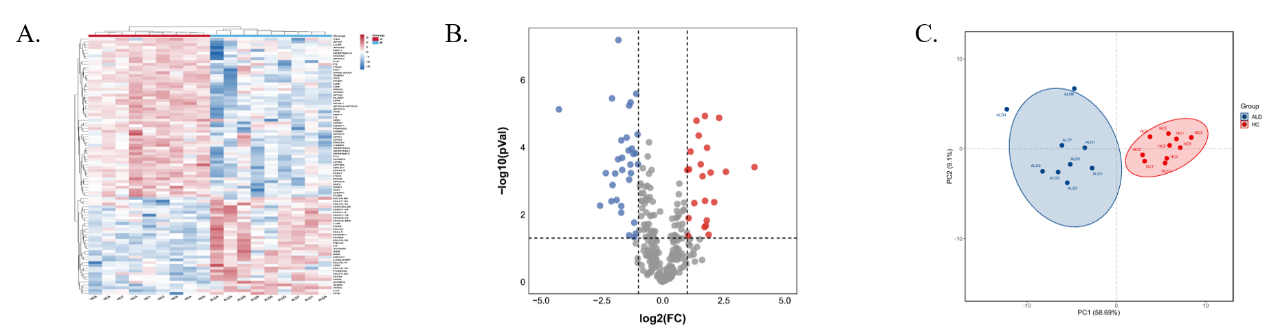


Supplementary Figure S1 Differential expressed proteins of clinical patients proteomic without abundant protein depletion. A.Heatmap represents gene expression trends in HC and ALD groups. B.Volcano plot shows the identified proteins of HC and ALD groups distribution. C.PCA shows two dimensionalities of HC and ALD groups according to differential proteins.


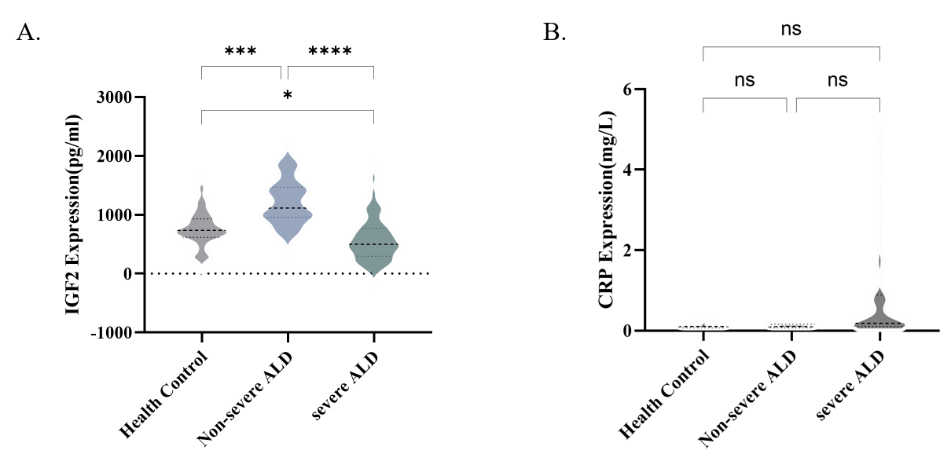
Supplementary Figure S2 Serum concentration of CRP in HC group, non-severe ALD group, and severe ALD group.

Supplementary Table S2 Characteristics of ROC curves for CRP

|  | **AUROC** | **95% CI** | **Sensitivity** | **Specificity** | ***P* value** |
| --- | --- | --- | --- | --- | --- |
| **HC-SA** | 0.7607 | 0.2953 to 0.6419 | 0.4643 | 1.0000 | 0.0669 |
| **HC-NSA** | 0.5200 | 0.1389 to 0.9011 | 0.8000 | 0.4000 | 0.9168 |
| **SA-NSA** | 0.7393 | 0.3581 to 0.7047 | 0.5357 | 1.0000 | 0.0926 |

Supplementary Table S3 Characteristics of CRP diagnostic models for severe ALD

|  |  | **AUROC** | **95%CI** | **Sensitivity** | **Specificity** | ***P* value** |
| --- | --- | --- | --- | --- | --- | --- |
| **Model E** | CRP-B2M | 0.9214 | 0.7280 to 0.9629 | 0.8929 | 1.0000 | 0.0031 |
| **Model F** | CRP-IGFBP3 | 0.8143 | 0.5294 to 0.8475 | 0.7143 | 1.0000 | 0.0272 |
| **Model G** | CRP-IGFALS | 1.0000 | 0.8794 to 1.000 | 1.0000 | 1.0000 | 0.0004 |
| **Model H** | CRP-B2M-IGFBP3 | 0.9286 | 0.7280 to 0.9629 | 0.8929 | 1.0000 | 0.0026 |

Supplementary Table S4 Characteristics of B2M quantity (ug/ml) in three groups

|  | **HC** | **Non-****severe ALD** | **Severe ALD** |
| --- | --- | --- | --- |
| **Mean** | 1.52 | 2.82 | 3.70 |
| **Median** | 1.50 | 2.00 | 2.60 |
| **First quartile (Q1)** | 1.35 | 1.70 | 2.25 |
| **Third quartile (Q3)** | 1.70 | 2.55 | 3.35 |
| **Minimum** | 1.20 | 1.60 | 1.40 |
| **Maximum** | 1.90 | 10.70 | 20.60 |

* *P*<0.001 of Non-severe ALD group compared to HC group based on *U* test.

*P*<0.001 of Severe ALD group compared to HC group based on *U* test.

*P*=0.023 of Severe ALD group compared to HC group based on *U* test.
